# Right subaxillary small incision on low-age and low-weight infants with congenital heart disease

**DOI:** 10.3389/fcvm.2024.1468673

**Published:** 2024-10-08

**Authors:** Xiaoyong Jing, Lun Li, Yongtao Wu, Zhiyi Wang, Lizhi Lv, Qiang Wang

**Affiliations:** Pediatric Heart Center, Beijing Anzhen Hospital, Beijing, China

**Keywords:** congenital heart disease, low-weight, low-age, right subaxillary small incision, sternal median incision

## Abstract

**Background:**

As a minimally invasive approach to treating congenital heart disease (CHD), the application of the right subaxillary small incision (RSSI) has been developing fast in its indication spectrum. However, RSSI's use is still quite challenging for the surgical treatment of the low-age and low-weight infant patient group.

**Objectives:**

To investigate the safety and efficacy of performing RSSI surgery, treating CHD for infants with low-age and low-weight.

**Methods:**

Between March 2010 and April 2023, Low-age (≤6 months) and low-weight (≤5 kg) infants with ventricular or atrial septal defect (ASD) as the main diagnosis were retrospectively included in this study. The infants were divided into the RSSI group and the median sternotomy (MS) group. Preoperative conditions, general surgical conditions, perioperative and postoperative outcomes, and long-term follow-up results were compared between the 2 groups.

**Results:**

The study included 783 cases. Of these, 434 were operated with MS, and 349 were operated with RSSI. A1:1 matched MS group (282 cases) and RSSI group (282 cases) were obtained after performing propensity score matching (PSM). Analysis of the 2 groups after PSM showed the differences in residual ASD/VSD, peak airway pressure, fraction of inspired oxygen/partial pressure of oxygen (PaO_2_/FiO_2_), and partial pressure of carbon dioxide (PaCO_2_) before return to the intensive care unit (ICU) and extubation were not statistically significant between groups, whereas mechanical ventilation time (*P* < 0.001), ICU stay (*P* < 0.001), and hospitalization time (*P* < 0.001) were lower in RSSI group; and the differences in perioperative complication outcomes were not statistically significant. Long-term outcomes revealed the rate of thoracic deformity was higher in the MS group.

**Conclusions:**

Based on the appropriate selection of patients, compared with MS, RSSI surgery for the treatment of low-age (≤6 months) and low-weight (≤5 kg) infants with CHD yields favorable therapeutic results without increasing surgical risks and causes less trauma, which deserves to be further promoted and applied.

## Introduction

Congenital heart disease (CHD) is currently the major birth defect disease. Relevant data point out that the global prevalence of CHD in live births is around 1% (1). Some of the CHD can be cured spontaneously, although most of them still need surgical intervention. Surgical treatment is the standard of care for correcting malformations and currently, the median chest incision is still the main approach for surgery.

Since the first case of right subaxillary small incision (RSSI) for the surgical correction of CHD was reported in 1994 (2), the indications for the treatment of CHD with an RSSI have been expanding, and the safety and therapeutic efficacy of this approach have been widely reported (3, 4). Compared with the median thoracic incision, this approach has a shorter incision, no bone injury, less blood loss, lower infection rate, and especially the scar is hidden under the axilla, which significantly reduces the patient's psychological trauma (5). Nonetheless, the relatively severe condition and incomplete lung development in low-weight infants (6) have largely limited the use of this minimally invasive procedure in this population. To our knowledge, there is a paucity of research concerning the application of the RSSI technique in infants of low-age and low-weight to date.

This study retrospectively investigates the real-world outcomes of RSSI surgery in the correction of low-age, low-weight infants with CHD, by evaluating the safety (mechanical ventilation time as the primary endpoint) and efficacy (residual VSD and/or ASD as the secondary endpoint) related parameters. With the findings of this study, we hope to provide a more accurate reference for future surgical decision-making in low-age and low-weight infants with CHD.

## Methods

### Ethical statement

The study was approved by the ethics committee of Beijing Anzhen Hospital [approval date: Feb. 2, 2024; approval code: (2024) Ke Lun Shen 1]. Due to the retrospective nature of this study, patient informed consent was waived.

### Study outcome endpoints

Postoperative mechanical ventilation time was used as the primary endpoint (safety endpoint) in this study, while septal residual VSD/ASD was evaluated as the secondary endpoint (efficacy endpoint).

### Sample size calculation

Statistical hypothesis: H0:xT−xC≥Δ;H1:xT−xC<Δ.

In above formulas: xT: Postoperative mechanical ventilation time in the RSSI group (experimental group); xC: Postoperative ventilator support time in the MS group (control group); Δ: Non-inferiority margin (set to 0 in this study).

Based on a previous retrospective study (6), the estimated postoperative mechanical ventilation time was 26.09 h (95% CI, 21.8–30.4) for the RSSI group and 28.04 h (95% CI, 25.5–30.6) for the MS group. Considering clinical experience, we assumed a postoperative mechanical ventilation time of 24 h for the RSSI group and 30 h for the MS group, with a pooled standard deviation of 15 h.

When the significance level is set at 0.05 (two-sided) and the power is set as 80%, with a 1:1 randomization ratio between the experimental and control groups, the required sample size is calculated as 198 subjects (99 in each group). Considering a 10% dropout rate, the final sample size is 220 subjects (110 in each group).

Sample size calculation formula: n={2[μ_(1−α)+ $\;{\mu \_(1-\beta )]}^{2}\sigma^{2}\}/[\Delta -(\mathrm{xT}-\;\mathrm{xC}){]}^{2}$.

Where: xT, xC, σ, and Δ are as defined above. µ represents the quantile of the standard normal distribution. α is the type I error rate (set to 0.05 for a two-sided test). β is the type II error rate (set to 0.2, corresponding to an 80% power).

### General information

A total of 783 consecutive low-weight (≤5 kg) and low-age (≤6 months) infants with ventricular septal defect (VSD) or atrial septal defect (ASD) as the primary diagnosis, who were treated surgically in the Pediatric Heart Center of Beijing Anzhen Hospital of Capital Medical University between March 2010 and April 2023, were included in this retrospective study. The lesions covered a wide range of common malformations such as patent foramen ovale/ASD, ductus arteriosus, right ventricular outflow tract stenosis/pulmonary valve stenosis, mitral/tricuspid valve insufficiency, partial anomalous pulmonary venous return, persistent left superior vena cava. The following conditions were excluded: neonatal surgery; secondary surgery, complex malformations such as tetralogy of Fallot/arterial arch constriction; and ≤1 month of age (as our center has not accumulated rich experience on neonatal surgery for this particular age group, infants under this age were excluded to avoid case selection bias). Of these patients, 434 were operated with median thoracotomy (MS) and 349 were treatment with RSSI approach.

For all subjects, whether to be treated via MS or RSSI was according to the surgeon's choice. For more details about surgeon's consideration, please refer to the Discussion section.

### Surgical method

For the MS group, the traditional surgical method was used, with the patient lying in the supine position and the back slightly elevated. The incision was made from the superior sternal fossa to the xiphoid process. Subsequently, the sternum was split in the middle, and the pericardium was suspended. Cardiopulmonary bypass was established as routine. Deformity correction was performed according to the patient's condition.

**Figure 1:**
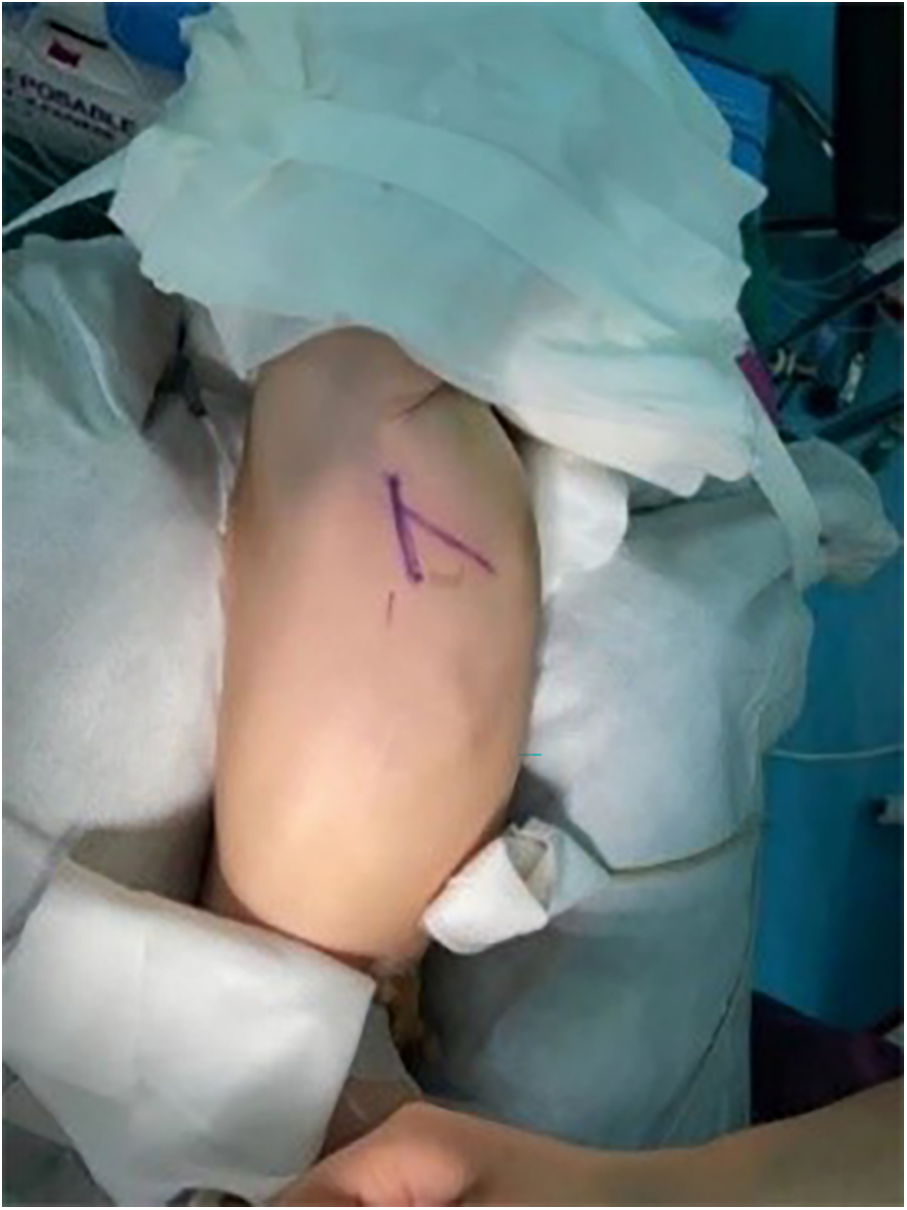
Right lateral small incision body position placement and line drawing.

For the RSSI group, after tracheal intubation under general anesthesia, airway pressure and breath sound on both sides was judged to be without abnormality. The infant was turned to the left lateral position and his/her position was fixed anteriorly and posteriorly. A subaxillary cushion (thickness: about 5 cm) and an under-the-head cushion were used to avoid excessive neck tension (Figure 1). The right arm was placed upward and outward to the head, and a cushion (thickness: about 4 cm) was put in the angle between the right neck and the right arm, for the purpose of keeping the angle at no less than 60°. The right arm was then fixed with a tape pad to prevent the right arm from moving during the operation and to avoid excessive stretching of the right arm. An incision (length: 3–6 cm) was made through the intersection of the 4th intercostal space and the mid-axillary line as the starting point, and the slightly posterior point to the intersection of the sixth rib and the anterior axillary line as the endpoint. The incision could be extended upward or downward slightly according to the surgeon's judgement. With the incision performed, the surgeon would then separate the muscles along the muscle gap of the pectoralis major and minor muscles to expose the intercostal space, and choose the 4th or the 5th intercostal space to enter the chest. During the incision, much care was taken to protect the long thoracic nerve and internal mammary artery, and the right lung was carefully blocked with wet gauze to the right posterior side to expose the pericardium and the right septal nerve. The pericardium was incised and hung up, and then the cardiopulmonary bypass was routinely established. The intracardiac anomalies were corrected accordingly.

### Data analysis

1. Preoperative data were collected and compared.
2. Surgical effects evaluation: perioperative indicators, respiratory function indicators and deformity correction effects were assessed. Vasoactive drug application was also assessed by calculating the vasoactive inotropic score (VIS) (7).
3. Long-term follow-up: parameters including thoracic deformity, respiratory tract susceptibility as well as growth and developmental status were analyzed.

### Study flow diagram

**Figure 2:**
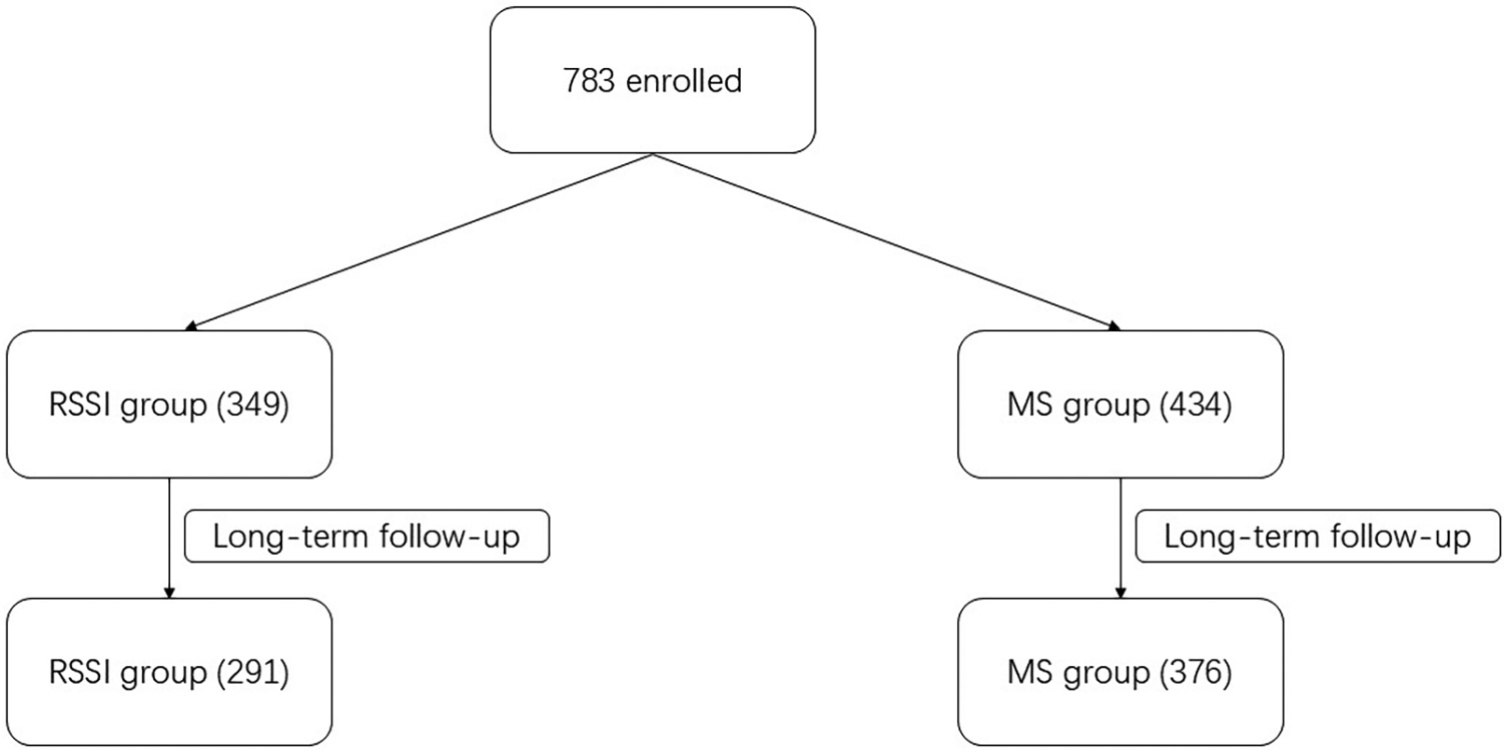
Study flow diagram.

About the flow diagram of our study, please refer to Figure 2.

### Statistical methods

The study data were statistically analyzed using SPSS 22.0. Measurement data were expressed as mean (standard deviation) by *t*-test, and count data were expressed as a percentage (%) by chi-squared test. *P* < 0.05 was regarded as a statistically significant difference. The propensity score matching (PSM) method was used to eliminate the potential confounding effect caused by unbalanced baseline parameters. Matched baseline variables included: age, weight, gender, and left ventricular ejection fraction. Regression results were presented in eight ways including unadjusted, multivariable adjusted, propensity score adjusted, propensity score matched, etc.

## Results

Eight surgeons, each with 15–40 years of practice experience, performed all the procedures included in this study. The preoperative data analysis of the infants in the 2 groups displays that the differences in age, weight, left heart size and pulmonary hypertension between the 2 groups were statistically significant (Table 1).

**Table 1:** Basic preoperative data (before PSM).

	RSSI group (*n* = 349)	MS group *n* = 434)	*P*-value
Age (day)	126.1 (33.5)	109.8 (36.8)	<0.001
Weight (kg)	4.75 (0.32)	4.53 (0.49)	<0.001
Female/Male	222/127	259/175	0.294
Left heart size (mm)	28.71 (3.15)	27.77 (3.86)	<0.001
Left ventricular ejection fraction (%)	70.3 (2.4)	70.3 (1.1)	0.965
Pulmonary hypertension (*n*)	254	348	0.015
History of pneumonia in the past half month before surgery (*n*)	92	47	<0.001

To exclude statistical errors due to differences in age and weight, the PSM method was conducted to generate 2 matched (ratio: 1:1) groups (post-PSM: each group with 282 subjects). After PSM (the infants after PSM had VSDs as their primary diagnosis, thus adding one more datum: the preoperative size of VSD; relevant data demonstrated in Table 2), there were no statistically significant differences between the 2 groups in terms of age and weight. Besides, there were no statistically significant differences in gender, VSD size, left heart size, or pulmonary hypertension. The proportion of patients with a history of preoperative pneumonia was higher in the MS group (*P* < 0.001).

**Table 2:** Basic preoperative data after PSM.

	RSSI group (*n* = 282)	MS group (*n* = 282)	*P*-value
Age (day)	121.0 (33.2)	116.0 (35.6)	0.091
Weight (kg)	4.7 (0.3)	4.7 (0.4)	0.052
Female/male	170/112	174/108	0.73
Left heart size (mm)	28.4 (3.2)	28.3 (3.7)	0.793
Left ventricular ejection fraction (%)	70.7 (5.3)	71.1 (5.3)	0.6
Preoperative VSD size (mm)	10.3 (2.7)	10.4 (3.0)	0.81
Pulmonary Hypertension (mmHg)	217	220	0.762
History of pneumonia half a month before surgery (*n*)	77	35	<0.001

Prior to PSM, VSD was the primary diagnosis in both groups, except for three cases of central secondary ASD in the RSSI group and one case of central secondary ASD combined with pulmonary valve stenosis in the MS group. There was no statistically significant difference in the major and combined malformations between the 2 groups. After PSM (Tables 3, 4), both groups had VSDs as the primary diagnosis (thus reducing the number the types of preoperative primary malformations by one: ASD, compared with pre-PSM), and the differences in the location of the VSDs and combined malformations were not statistically significant (post-PSM, due to the exclusion of some of the infants, the number of the types of combined malformations was reduced by two in comparison with pre-PSM).

**Table 3:** Major preoperative deformities after PSM.

	RSSI group (*n* = 282)	MS group (*n* = 282)	*P*-value
Perimembranous VSD	240	235	0.564
Subarterial VSD	17	24	0.256
Intracristal VSD	12	10	0.664
Perimembranous to subarterial VSD	2	2	1.0
Multiple small perimembranous combined muscular VSDs	8	4	0.243
Muscular VSD	2	5	0.447
Type of VSD: Endocardial cushion defect	1	2	1.0

**Table 4:** Preoperative combined deformities after PSM.

	RSSI group (*n* = 282)	MS group (*n* = 282)	*P*-value
ASD	75	86	0.305
Patent foramen ovale	110	108	0.863
Patent ductus arteriosus	22	30	0.244
Persistent left superior vena cava	16	22	0.314
Right ventricular outflow tract stenosis	10	8	0.104
Mitral valve insufficiency	6	12	0.151
Tricuspid valve insufficiency	6	8	0.588
Subaortic membrane	4	4	1.0
Pulmonary valve stenosis	1	2	1.0

The results of perioperative surgical and respiratory function outcomes before PSM are listed in (Supplementary Tables S1, S2), which present the mechanical ventilation time, ICU time and hospitalization time were shorter in the RSSI group (all *Ps* < 0.001) while the PaO_2_/FiO_2_ was slightly higher in the RSSI group (only for before extubation; *P* = 0.012). The results of perioperative and respiratory function indexes after PSM are recorded in Tables 5, 6. The results demonstrate the differences in peak airway pressure, PaO_2_/FiO_2_ and PaCO_2_, before returning to ICU and extubation, were not statistically significant between the 2 groups while mechanical ventilation time, ICU time and hospitalization time were shorter in the RSSI group (all *Ps* < 0.001).

**Table 5:** Comparison of perioperative data after PSM.

	RSSI group (*n* = 282)	MS group (*n* = 282)	*P*-value
Operation time (min)	157.1 (28.1)	158.2 (72.2)	0.867
Cardiopulmonary bypass time (min)	70.3 (21.1)	67.2 (22.3)	0.099
Aortic cross-clamping time (min)	39.4 (13.5)	37.8 (25.4)	0.372
Intraoperative drainage volume (ml)	56.2 (16.9)	56.2 (23.3)	0.98
Postoperative drainage volume on the day of operation (ml)	111.9 (46.5)	127.4 (58.9)	<0.001
Mechanical ventilation time (h)	48.9 (44.3)	57.7 (48.6)	<0.001
ICU treatment time (days)	6.4 (5.1)	8.8 (5.1)	<0.001
Hospitalization time (days)	11.1 (6.6)	14.3 (7.6)	<0.001
Postoperative left heart size (mm)	23.0 (17.1)	22.5 (2.5)	0.606
Postoperative cardiac function status (%)	65.9 (7.1)	67.9 (6.6)	<0.001
VIS	8. 51 (3.24)	7.83 (3.35)	0.025

VIS, vasoactive inotropic score.

**Table 6:** Comparison of respiratory function after PSM.

	RSSI group (*n* = 282)	MS group (*n* = 282)	*P*-value
PaO_2_/FiO_2_ upon returning to ICU	330 (120)	340 (120)	0.323
PaO_2_/FiO_2_ before extubation	290 (120)	280 (120)	0.323
PaCO_2_ upon returning to ICU (mmHg)	40.7 (10.0)	40.8 (6.8)	0.924
PaCO_2_ before extubation (mmHg)	37.5 (4.6)	38.6 (18.3)	0.332
Peak airway pressure upon returning to ICU (mmHg)	16.6 (2.2)	16.8 (2.2)	0.297
Peak airway pressure before extubation (mmHg)	16.3 (2.1)	16.0 (1.9)	0.16

Postoperative residual ASD/VSD's difference between 2 groups were not statistically significant. Also, assessing the conditions related to perioperative complications in the 2 groups of infants indicates that the difference in the condition of complications between the 2 groups was not statistically significant. Relevant data shown in Supplementary Table S4 (pre-PSM) and Table 7 (post-PSM).

**Table 7:** Comparison of perioperative efficacy and complications after PSM.

	RSSI group (*n* = 282)	MS group (*n* = 282)	*P*-value
Residual VSD	19	24	0.428
Death	0	2	0.479
Reoperation	1	1	1.0
Pericardial effusion openings	1	3	0.616
Poor wound healing and incision cleaning	1	5	0.218
Implantation of pacemaker	1	1	1.0

The long-term follow-up (tele-follow-up) was conducted in February 2024. Due to substantial economic and cultural variations, a considerable portion of participants could not be followed up successfully, despite much efforts made. During the tele-follow-up, all subjects were asked to send videos and/or photographs of the chest appearance and were questioned, to evaluate if there was any complication. Follow-up time: 79.7 ± 7.1 months (range: 7–127 months). The results for both groups after excluding data relating to loss to follow-up and discharge on patient's request are provided in Table 4 (pre-PSM; Supplementary Material) and Table 8 (post-PSM). A chi-squared test was performed on the long-term follow-up data, and the result after PSM show the rate of thoracic deformity was higher (*P* = 0.001) in the MS group (7.4%) than in the RSSI group (1.4%), and the differences in respiratory disease susceptibility and growth were not statistically significant between the 2 groups.

**Table 8:** Long-term follow-up outcome (after PSM).

	RSSI group (*n* = 282)	MS group (*n* = 282)	*P*-value
Thoracic deformity	4 (1.4%)	21 (7.4%)	0.001
Respiratory tract susceptibility	6 (2.1%)	6 (2.1%)	0.764
Growth and developmental deviation	48 (17.0%)	52 (18.4%)	0.740

## Discussions

The technique of small incision thoracotomy surgery can be categorized into: lower mini-sternotomy, right anterior mini-thoracotomy and RSSI surgery (8). RSSI surgery has been developed for more than two decades, and its safety and efficacy have been confirmed by several studies (9–11). Despite this, the relative severity of the condition of infants with low-age and low-weight, coupled with the difficulty of lung protection, has led to most being treated with MS, while the use of the RSSI has been limited. Our study provided retrospective data and related analysis of RSSI's safety and efficacy on this specific patient group.

In this study, the preoperative history of pneumonia one month before the surgery was higher in the RSSI group (pre- and post-PSM). The reason may mainly be related to: A. the preference for RSSI by the infants' parents as well the subsequent evaluation and decision by the surgeon; B. for the infants aiming to be operated using RSSI, it is generally advisable to wait until the infant is older. C. pneumonia might occur while waiting, then the RSSI surgery will be performed once pneumonia is cured. Also before PSM was performed, the MS group was younger and weighed less compared to the RSSI group, owing to the surgeon's choice: since the RSSI for younger and lower-weight infants has not been fully promoted, the surgeons are overall more inclined to select more confident cases for RSSI choice. Younger age and lower weight may lead to the more frequent combination of pulmonary hypertension in the MS group before PSM conducted.

In patients with combined ASD, patent foramen ovale, or tricuspid valve insufficiency, the right atrium is more adequately exposed through a right-sided incision into the chest, and therefore there is no increase in the difficulty or risk of the procedure in these patients. In this study, one patient with anomalous pulmonary venous return had an anomalous return of the right inferior pulmonary vein into the right atrium, which did not increase the difficulty of the procedure. However, in patients with severe pulmonary hypertension and a mismatch between the size of the left heart and the size of the VSD, it is recommended to exclude the possibility of partial anomalous pulmonary venous drainage (supracardiac). In patients with anomalous pulmonary venous return whose returning port is distant from the atria, RSSI is not recommended. In infants with combined mitral valve insufficiency, ductus arteriosus, localized stenosis of the right ventricular outflow tract or pulmonary valve stenosis, subaortic membrane, or persistent left vena cava, the difference in operative time between the 2 groups in this study, before and after PSM, was not statistically significant, although it would have increased the operative time. Also, it is worth noting that one of the infants in this study had a mesocardia with dextroversion, and the RSSI was chosen to provide a more definitive exposure of the aorta and right atrium, with favorable results in repairing the VSD via the tricuspid valve.

The difference in aortic cross-clamp time, cardiopulmonary bypass time, and operative time between the 2 groups of infants before and after PSM was not statistically significant in both groups. This suggests that after cardiopulmonary bypass has been established, the difficulty of surgical manipulation within the heart is comparable in infants of low-age and low-weight. Postoperative drainage was statistically higher in the median-opening group (pre- and post-PSM), which may be related to the fact that splitting the sternum requires disruption of the bone marrow cavity for blood leakage. Compared with the MS group, the ventilator time, ICU time, and hospitalization time were statistically shorter in the RSSI group (before and after PSM). This is consistent with previous reports (12, 13), except the subjects in our study were younger infants with lower body weight This should be related to the fact that the right-side incision did not saw through the sternum, which ensured the integrity of the thorax and was relatively less traumatic.

Given that the RSSI needs to be made from the right side of the chest, it inevitably needs to have compression or friction on the right lung, especially for infants of low-age and low-weight at the same time, who suffer interstitial pulmonary congestion and worse condition, which makes performing RSSI more challenging. Among all the infants in the RSSI group, we paid special attention to lung protection. Previous data have noted that alveolar collapse is prolonged during RSSI surgery, thereby potentially impairing respiratory function in children (14). In this study, before PSM, the differences in postoperative peak airway pressure and PaCO_2_ between the 2 groups were not statistically significant, and the PaO_2_/FiO_2_ before extubation was slightly lower in the MS group, which may be related to the smaller age and weight. The differences in postoperative peak airway pressure, PaCO_2_, and PaO_2_/FiO_2_ between the 2 groups of patients after PSM were not statistically significant, which proved that there was no significant difference in both pulmonary ventilation and gas exchange function between the 2 groups of patients after surgery, and also demonstrated that the RSSI did not increase the probability of lung injury with proper lung protection strategies.

When considering and comparing the treating efficacy of these 2 groups: there was no significant difference in the incidence of residual VSD (pre- and post-PSM), which indicates operation with RSSI approach can achieve favorable intracardiac malformation exposure without obvious increasing the risk/difficulty of procedural maneuver. The difference on perioperative complications (pre- and post-PSM) between the 2 groups was not statistically significant, neither. There was a trend of a higher probability of poor wound healing requiring debridement and pericardial effusion in the MS group (pre- and post-PSM; the difference was not statistically significant), which was considered to be related to: infants with MS had greater respiratory sternal mobility and higher tension on the tissues incised, which made them susceptible to effusions that could affect wound healing, while infants with a right-sided incision after pericardiotomy the pericardium could be communicated with the right side of the thorax, which made them less susceptible to pericardial effusion.

**Figure 3:**
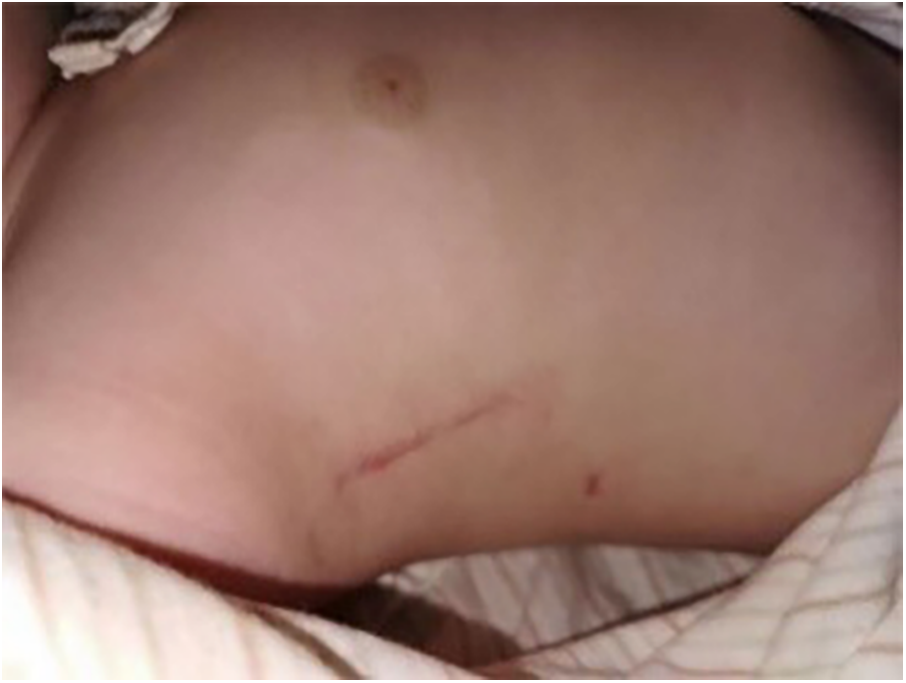
Incision at 7 days postoperatively.

Takashi et al. (15) pointed out significant postoperative scoliosis occurred in children under one year of age who underwent surgery through the median sternal approach, and the mechanism of occurrence may be related to mechanical injury to the ribs caused by median thoracotomy. In addition to the possibility of scoliosis, the sternum must be split due to median sternotomy, which disrupts the continuity of the sternum and leads to thoracic deformity. As the long-term follow-up results of this study showed (pre-PSM & post-PSM), the incidence of thoracic deformity in the MS group was significantly higher than that in the RSSI group. The incision performance of a case in the RSSI group before discharge is illustrated in Figure 3.

Nevertheless, compared with the MS approach, we should also note the RSSI technique still has a few shortcomings. In this study, the LVEF of the RSSI group was slightly lower than that of the MS group in the perioperative period, while VIS in the perioperative period (pre-and post-PMS for LVEF and VIS) were slightly higher in the RSSI group than that of the MS group. This suggests that, in order to better expose the target location of the VSD in the intraoperative period, the RSSI group needed to pull on the heart more significantly thus possibly affecting postoperative cardiac function.

The incision choice regarding RSSI should be decided after careful evaluation of the infant's age, weight, and complexity of the disease so that the safety of the operation can be guaranteed. We do not recommend RSSI surgery as the first choice for patients with combined pneumonia and heart failure, patients with large PDA, large myocardium septal defects near the apex, and patients with pulmonary dysplasia. It is also noted that if the preoperative airway pressure was too high, and there is no significant improvement after adjustment by the anesthesiologist, then we suggest avoid the RSSI approach. And last but not least, the surgical team should master the MS surgery very well before performing any RSSI surgical treatment.

## Limitations

This study is a retrospective observational cohort study. Although we applied the PSM method, there may still be potential confounding factors which were not considered, such as: surgeons’ practicing experience, potential diseases, preoperative medication, The reason for this was mainly due to the subjects' main diagnoses were VSD or ASD which were considered as simple CHD, which making us consider there were no significant differences on these factors.

## Conclusions

The results of this study provide early evidence that in infants with low-age (≤6 months) and low-weight (≤5 kg) with CHD, based on proper selection of indications, the application of RSSI is as safe and effective as that observed with the MS approach, while RSSI approach causes less trauma, which shows great promise for broad clinical application.

## Data Availability

The original contributions presented in the study are included in the article/Supplementary Material, further inquiries can be directed to the corresponding author.

## Supplementary material

Supplementary Video S1 Skin incising and performing cardiopulmonary bypass.

Supplementary Video S2 Repair of VSD.

Supplementary Video S3 Chest closing.
