# Supplementary material for: Right subaxillary small incision on low-age and low-weight infants with congenital heart disease
Source: Front Cardiovasc Med. 2024 Oct 8;11:1468673. doi: 10.3389/fcvm.2024.1468673 (PMC11493727; doi:10.3389/fcvm.2024.1468673)
Supplement: Supplementary file 4 [file Table1.pdf]

Supplementary Table 1: Comparison of perioperative surgical outcome data (before PSM)

	RSSI group (n=349)	MS group (n=434)	P-value
Operation time (min)	157.3 (68.6)	159.3 (29.3)	0.622
Cardiopulmonary bypass time (min)	70.9 (21.9)	68.9 (21.4)	0.209
Aortic cross-clamping time (min)	38.9 (13.1)	38.2 (22.8)	0.635
Intraoperative drainage volume (ml)	55.3 (23.0)	55.76 (17.2)	0.837
Postoperative drainage volume on the day of operation (ml)	111.1 (43.5)	124.4 (50.6)	<0.001
Duration of mechanical ventilation (h)	48.3 (40.2)	70.9 (67.5)	<0.001
ICU treatment time (min)	6.17 (4.73)	8.50 (5.10)	<0.001
Hospitalization time (min)	10.67 (6.23)	13.53 (7.29)	<0.001
Postoperative left heart size (mm)	22.67 (2.42)	22.51 (14.36)	0.824
Postoperative cardiac function status (%)	65.6 (7.0)	67.9 (6.6)	<0.001

VIS	8. 37 (3.35)	7.82 (3.45)	0.031

VIS: vasoactive inotropic score

Supplementary Table 2: Comparison of perioperative respiratory function (before PSM)

	RSSI group (n=349)	MS group (n=434)	P-value
PaO_2/FiO_2 upon returning to ICU	321 (121)	330(119)	0.297
PaO_2/FiO_2 before extubation	292 (121)	270(122)	0.012
$PaCO_2$ upon returning to ICU (mmHg)	40.89 (9.26)	40.78(6.52)	0.852
$PaCO_2$ before extubation (mmHg)	37.84 (4.67)	38.36(15.49)	0.518
Peak airway pressure upon returning to ICU (mmHg)	16.6 (2.1)	16.9 (2.3)	0.327
Peak airway pressure before extubation (mmHg)	16.2 (2.2)	16.0 (2.0)	0.13

Supplementary Table 3: Comparison of outcomes of perioperative efficacy and complication (before PSM)

	RSSI group (n=349)	MS group (n=434)	P-value
Residual ASD	0	0	N/A
Residual VSD	23	28	0.995
Death	0	4	0.241
Reoperation	2	1	0.836
Pericardial window for pericardial effusion	1	4	0.524
Poor wound healing and incision cleaning	1	6	0.225
Implantation of pacemaker	1	1	1

Supplementary Table 4: Long-term follow-up outcome (before PSM)

	RSSI group (n=291)	MS group (n=376)	P-value
Thoracic deformity	4 (1.4%)	25 (6.6%)	0.001
Respiratory tract susceptibility	7 (2.4%)	7 (1.9%)	0.627
Growth and developmental deviation	49	58	0.622
